# *Streptococcus pneumoniae* Attenuated Strain SPY1 with an Artificial Mineral Shell Induces Humoral and Th17 Cellular Immunity and Protects Mice against Pneumococcal Infection

**DOI:** 10.3389/fimmu.2017.01983

**Published:** 2018-01-11

**Authors:** Xinyuan Zhang, Jingjing Cui, Yingying Wu, Hong Wang, Jian Wang, Yulan Qiu, Yunjun Mo, Yujuan He, Xuemei Zhang, Yibing Yin, Wenchun Xu

**Affiliations:** ^1^Key Laboratory of Laboratory Medical Diagnostics Designated by the Ministry of Education, School of Laboratory Medicine, Chongqing Medical University, Chongqing, China; ^2^Department of Clinical Laboratory, Chongqing Hospital for Women and Children, Chongqing, China

**Keywords:** *Streptococcus pneumoniae*, live attenuated vaccine, biomineralization, thermal stability, immunogenicity

## Abstract

*Streptococcus pneumoniae* is a major pathogen leading to substantial morbidity and mortality in children under 5 years of age. Vaccination is an effective strategy to prevent *S. pneumoniae* infection. SPY1 is a pneumococcal vaccine candidate strain obtained in our previous study. To improve its stability and immunogencity, in this study, we constructed the SPY1Δ*lytA* strain that lacks autolysin activity and was coated with an artificial exterior surface calcium phosphate shell by *in situ* mineralization. The resulting strain SPY1Δ*lytA*CaPi displayed enhanced thermal stability enabling storage at 37°C for 1 week. Furthermore, mucosal and subcutaneous immunization with the SPY1Δ*lytA*CaPi strain induced better protective effects than SPY1Δ*lytA* in anti-colonization after challenging with 19F and anti-invasion by D39 in mice. Subcutaneous immunization with SPY1Δ*lytA*CaPi elicited higher IgG level while mucosal immunization primarily elicited an immune response which is supposed to be related to Th17 cells. Taken together, the mineralized strain may be a promising candidate for an attenuated *S. pneumoniae* vaccine.

## Introduction

*Streptococcus pneumoniae* is a major cause of pneumonia, otitis media, sepsis, and meningitis in children and the elderly (1). Vaccination is an effective strategy to prevent *S. pneumoniae* infection, the 23-valent pneumococcal polysaccharide vaccine and protein-conjugate vaccines (PCV) are currently in use including PCV7, PCV9, PCV10, and PCV13 (2–4). However, these vaccines have some disadvantages including weak immunogenicity, limited serotype coverage, serotype replacement, and high cost (5, 6). Therefore, developing a new and more ideal pneumococcal vaccine has become an important goal.

*Streptococcus pneumoniae* vaccine development includes whole-cell, polysaccharide, polysaccharide conjugate, as well as subunit protein vaccines. *S. pneumoniae* is highly diverse genetically and can rapidly change itself by recombination. A protein that is not essential for viability but significant in immunological recognition by the host can be altered or eliminated (*pspA* and *psrP*) (7). Candidate vaccines combining several common proteins may reduce the possibility that pneumococcus would readily evolve to evade immunity induced by vaccination. Generally, soluble antigen vaccines tend to lead host immune tolerance while bacteria-like particles including diversity antigens and agonists of toll-like receptors could activate multiple Toll-like receptors to induce stronger immune responses (8). Therefore, an attenuated live vaccine would be an ideal candidate to protect against pneumococcal infection.

SPY1 is a live attenuated strain of *S. pneumoniae* with significantly reduced amounts of capsular polysaccharide and pneumolysin (9). SPY1 can induce Th1/Th2/Th17 immune responses and protect mice against pneumococcal infection (10). SPY1 also offer better protection effect than the commercial 23-valent polysaccharide vaccine and therefore is a candidate vaccine strain (9). However, because of the weak immunogenicity of SPY1, it must be given with an adjuvant such as cholera toxin (CT) that is toxic for humans (11).

For vaccine, maintaining the cold chain is critical for adequate bioactivity. Despite preservation at low temperature, nearly half of the amount of vaccine was estimated to be abandoned around the world due to heat inactivation. And the cost of refrigeration contributes to about 80% of the cost of vaccine (12). Biological mineralization technology has been successfully used to improve thermal stability of living organisms (13–19). This technique involves the use of calcium phosphate to form an eggshell-like exterior that has improved the thermal stability and immunogenicity of viruses and yeast (17–19). A vaccine virus treated in this manner can be stored at 26°C for more than 9 days and at 37°C for 1 week (19). However, there are no reports of biomineralization used for live bacterial vaccines. In order to improve the thermal stability of SPY1 and further enhance its immunogenicity, this study focused on the *in situ* mineralization of SPY1 and evaluated its immune protection effects.

## Materials and Methods

### Mice

6- to 8-week-old C57BL/6 female mice were obtained from the animal center of Chongqing Medical University. Mice were kept under specific pathogen-free conditions at the animal centers of Chongqing Medical University during the time of the experiments.

### Bacteria Strains

*Streptococcus pneumoniae* strain NCTC 7466 (D39, serotype 2) was obtained from the National Collection of Type Cultures (NCTC; London, United Kingdom). *S. pneumoniae* clinical isolates CMCC 31693 (serotype 19F) were obtained from the National Center for Medical Culture Collections (CMCC; Beijing, China). The *S. pneumoniae* SPY1 strain used in this study was obtained previously and stored in 20% glycerol at −80°C (10). All *S. pneumoniae* strains were grown on Columbia sheep blood agar plates or in casein-based medium with yeast extract (C+Y medium) at 37°C under 5% CO_2_ atmosphere. Zeta potential were measured by Malvern Zetasizer when SPY1 and SPY1Δ*lytA* were suspended in sterility phosphate-buffered saline (PBS) at pH 7.2.

### SPY1Δ*lytA* Strain Construction

We constructed an attenuated *S. pneumoniae* strain SPY1Δ*lytA* by insertional inactivation. The plasmid pEVP3 were constructed with a *lytA* homologous arm was transformed into SPY1 and intergrated into the *lytA* gene by homologous recombination (20). Primers *lytA*-ins F and *lytA*-ins R were designed by Primer Premiers 5 according to the sequence of the *S. pneumoniae* D39 *lytA* gene (Table 1). The *lytA*-ins gene was integrated into the plasmid pEVP3 (21) and *pEVP3-lytA*-ins was inserted into the SPY1 *lytA* gene using insertional inactivation (20). The SPY1Δ*lytA* mutant strain was screened on blood plates containing erythromycin (0.25 µg/ml) and chloramphenicol (2.5 µg/ml). PCR were used to confirm the mutation in chloramphenicol resistant isolates (Table 1). To evaluate the production of LytA protein in SPY1Δ*lytA* mutant strain, the expression of LytA was analyzed by Western blotting with anti-LytA sera.

**Table 1:** The primers of amplification and identification of SPY1Δ*lytA* mutant strain.

Primer	Sequence (5′→3′)	Length (bp)
*lytA*-ins F	GAAGATCTAGATTTGCCTCAAGTCGG	421
*lytA*-ins R	CCCCCGGGAGGGTCAACGTGGTCTGA	
*lytA* Fexp BamH I	GGGGATCCATGGAAATTAATGTGAGTAAAT	6,723
*lytA* Rexp Xho I	CCGCTCGAGTTTTACTGTAATCAAGCCATC	
*pEVP3* F	ATCTCAGTTCGGTGTAGGTC	1,068
*pEVP3* R	TTATTGGGATAAGTTAGAGCC	
*ply* F	ATGGCAAATAAAGCAGTAAATGACT	1,416
*ply* R	CTAGTCATTTTCTACCTTATCCTCT	

### Biomineralization of SPY1Δ*lytA*

The SPY1Δ*lytA* mutant was cultured in C + Y liquid medium to mid-exponential growth phase (OD_620_ = 0.4–0.5, 1.5 × 10^8^ CFU/mL) and washed twice with 0.9% NaCl by centrifuging at 12,000 rpm for 5 min. The cell density was then adjusted to 1 × 10^9^ CFU/mL in normal saline. The bacterial suspension was added to 5 ml of a 10 mM CaCl_2_ solution and placed at 4°C for 1 h. The mixture was then centrifuged 10–15 min at 2,000 rpm and the supernatant was removed. The pellets were suspended with equal volumes of 4, 6, and 10 mM Na_2_HPO_4_ (pH 6.8) and centrifuged 5 min at 3,000 rpm. The pellets were washed and suspended in sterilize saline. Bacteria were visualized using scanning electron microscope (SEM, Model S-3000N, Hitachi, Japan).

### Immunization and Challenge

SPY1 and SPY1Δ*lytA* were grown at 37°C in 5% CO_2_ in C+Y medium to approximately 1.5 × 10^8^ CFU/ml. The cells were collected by centrifugation, washed twice, and suspended in sterile PBS. For mucosal immunization, the mice were randomly divided into six groups: PBS, CT (Sigma-Aldrich), SPY1, SPY1Δ*lytA*, SPY1Δ*lytA* + CT, and SPY1Δ*lytA*CaPi. Similarly, we divided the mice into six groups for subcutaneous immunization using: PBS, alum adjuvant (alum; Thermo Fisher Scientific), SPY1, SPY1Δ*lytA*, SPY1Δ*lytA* + alum, and SPY1Δ*lytA*CaPi. The experimental groups were vaccinated either subcutaneously with 100 µl SPY1 (l × 10^8^ CFU) mixed with equal volume of alum adjuvant or *via* the mucosa with SPY1 (l × 10^8^ CFU) plus 1 µg CT in 30 µl PBS. For subcutaneous immunization, mice were vaccinated three times over 2 weeks, whereas for mucosal immunization, mice were immunized once per week for four consecutive weeks. Serum samples were collected 1 week after the final immunization. Blood samples were collected from tails. All samples were stored at −80°C.

For the colonization model, 2 weeks after the last vaccination, mice were anesthetized and challenged with l × 10^8^ CFU of pneumococcal strain CMCC 31693 (serotype 19F) and sacrificed 72 h later. CFU counts in nasal washes and lung homogenates were determined as previously described (22). For the lethal intranasal challenge model, C57BL/6 mice were anesthetized and then inoculated intranasally with pneumococcal strain NCTC 7466 (D39, serotype 2; l × 10^8^ CFU) in 30 µl of PBS. Survival was monitored for 21 days.

### Antibody Titers and IgG Isotype Determinations

Protein concentrations were determined by BCA protein assay kit (Beyotime Institute of Biotechnology, Shanghai, China) and adjusted to equal protein concentrations. For measurement of antigen specific IgG titers, antibody levels were determined by using 96-well plates coated with SPY1 as previously described (22, 23).

### Cytokine Measurements

One week after the final immunization, mouse splenocytes were isolated (24) and transferred into 24-well tissue culture plates (2 × 10^6^ cells/well) in 1 ml of DMEM with 10% fetal calf serum (HyClone). Then, cells were treated for 72 h with Concanavalin A (5 mg/ml, Sigma, St. Louis, MO, USA) or 70% ethanol-killed SPY1 (equivalent to 10^7^ CFU/ml). Cytokines IFN, IL-4, IL-10, and IL-17A in the culture supernatants were determined using commercial kits (Biolegend, CA, USA) according to the manufacturer’s recommendations.

### Statistical Analysis

Data were compared using Student’s *t* test or a Mann–Whitney *U* test. Survival rates were analyzed with a log-rank test. Differences at a *P* value of <0.05 were considered significant. Statistical analysis was performed using GraphPad Prism, version 5.

## Results

### SPY1Δ*lytA* Strain Construction

An interesting characteristic of *Streptococcus pneumoniae*, autolysis, is highly related to LytA protein (25). In order to avoid bacterial autolysis during mineralization, non-autolysis SPY1 strain (SPY1Δ*lytA*) was constructed by insertional inactivation method.

**Figure 1:**
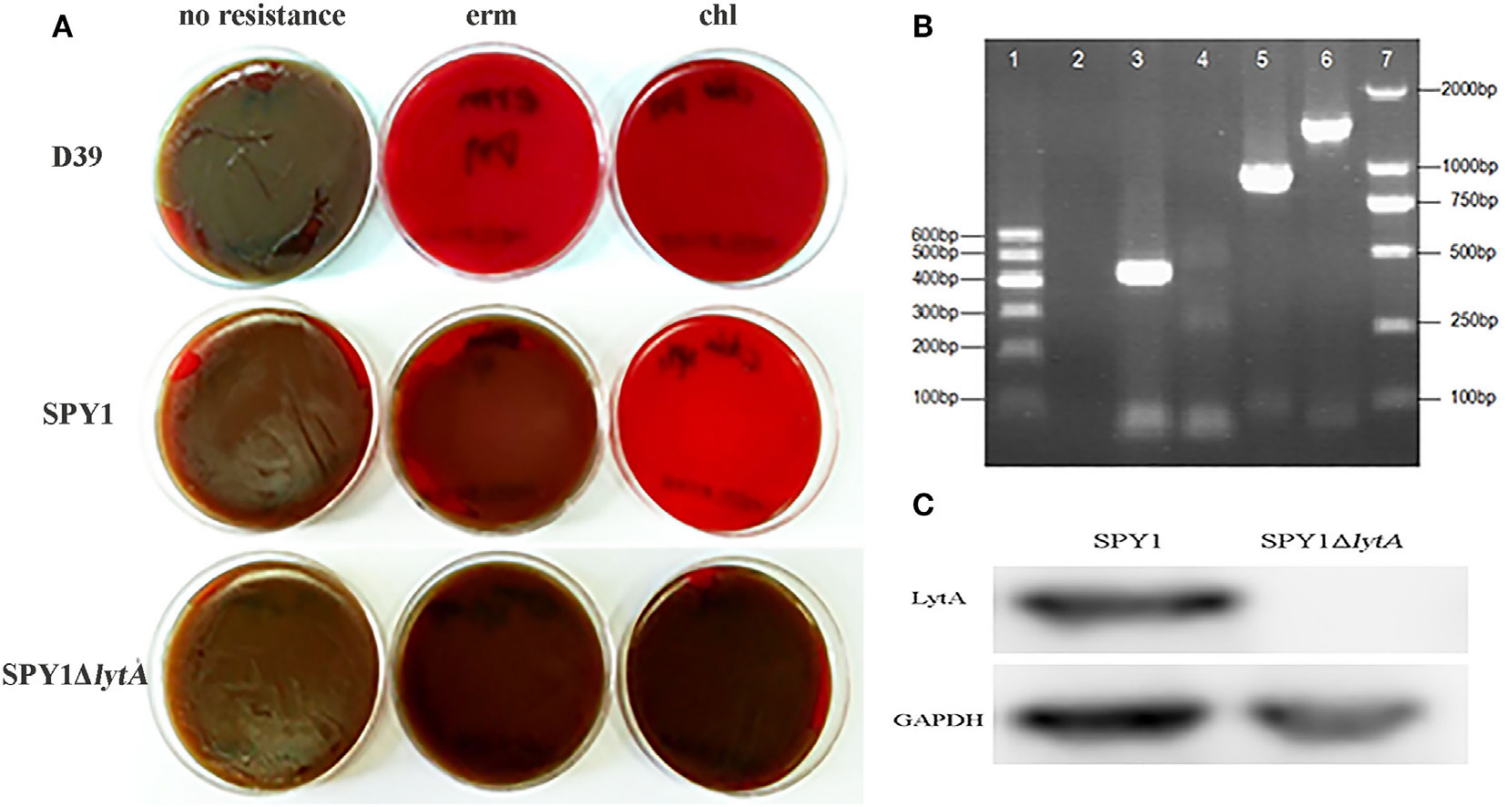
Construction of SPY1Δ*lytA*. **(A)** The antibiotic-resistance of constructed strains. *Streptococcus pneumoniae* strain D39, SPY1, and SPY1Δ*lytA* was seeded on blood agar plates supplemented erythromycin (erm) and chloramphenicol (chl) followed by 16 h incubation at 37°C under 5% CO_2_ atmosphere. **(B)** PCR products gel electrophoresis determination of plasmid integration. From left to right: 600 bp DNA marker (lane 1); Negative control, no template DNA (lane 2); *lytA*-ins (lane 3); *lytA* (lane 4); *pEVP3* vector fragment (lane 5); *ply* (lane 6); 2,000 bp DNA marker (lane 7). **(C)** Detection of LytA protein by western blotting.

Due to the erythromycin resistance of SPY1 and CAT gene from the introduced pEVP3 plasmid, blood agar plates supplemented with appropriate antibiotics were used to screen out the correctly constructed mutant strain SPY1Δ*lytA* (Figure 1A). Besides, SPY1Δ*lytA* strain was authenticated with PCR. The amplified fragment *lytA-*ins (lane 3) refers to the correct insertion of *lytA* homologous arm into the SPY1 strain. No *lytA fragments* were amplified *(lane 4) and* amplified pEVP3 fragment (lane 5) indicated that the pEVP3 vector was integrated into SPY1 chromosome and disrupted l*ytA* gene (Figure 1B). Western blot revealed that LytA was knocked out in SPY1Δ*lytA* (Figure 1C). In conclusion, LytA has been silenced in the SPY1Δ*lytA strain*.

### SPY1Δ*lytA* Strain Lacks Autolysis Ability

**Figure 2:**
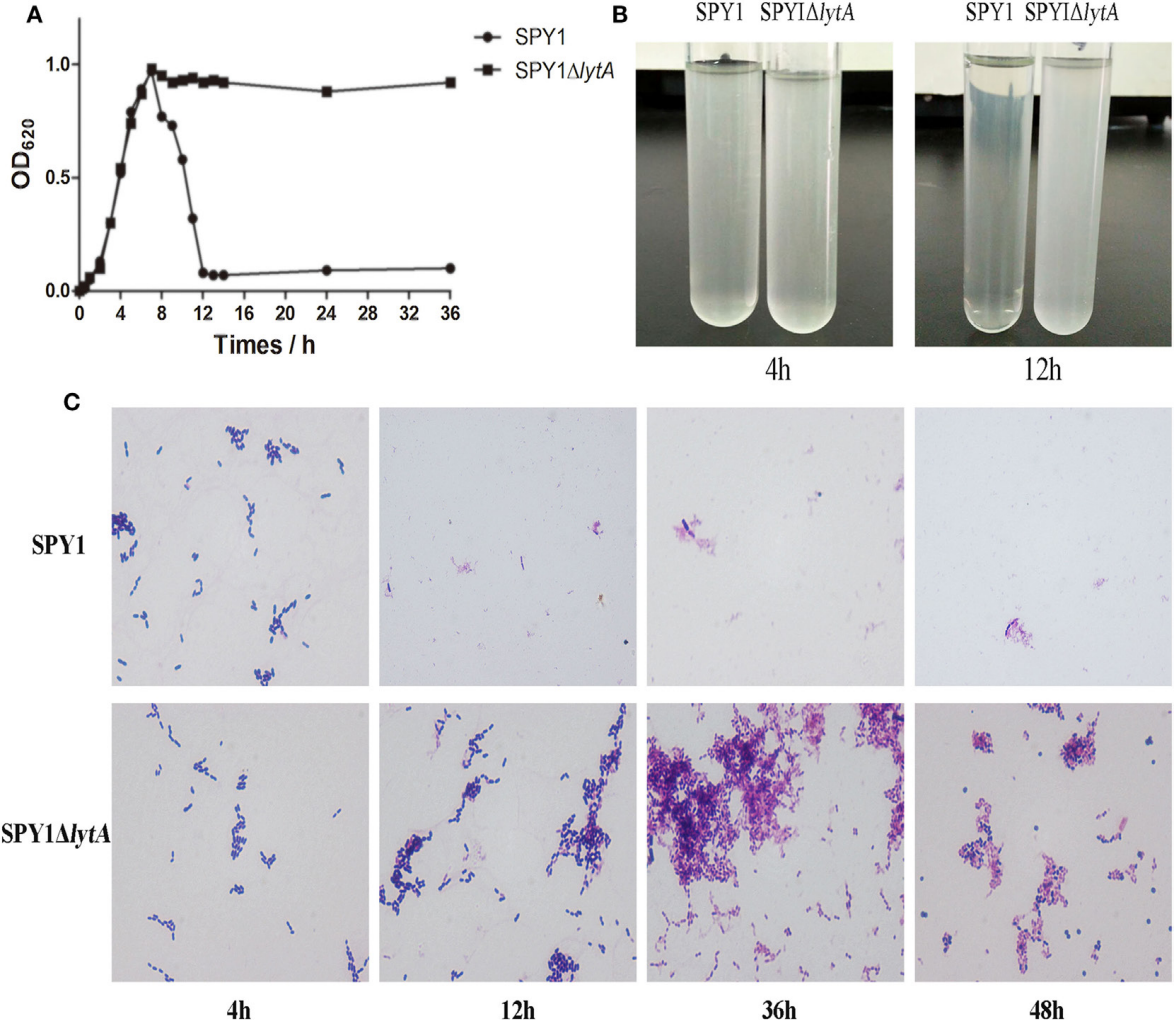
Growth and autolytic characteristics of strain SPY1Δ*lytA*. **(A)** Growth curve of SPY1 and SPY1Δ*lytA* in C + Y medium at 37°C. **(B)** Appearance of SPY1 and SPY1Δ*lytA* in C + Y medium at 4 and 12 h. **(C)** Morphology of Gram stained SPY1 and SPY1Δ*lytA* after incubation at 37°C for the indicated times.

Autolysis is an important feature of *S. pneumoniae* and LytA protein is essential to it (25). After *lytA* inactivation, the autolysis ability was measured. During culturing in C + Y medium, the OD_620_ of SPY1 increased gradually to about 1.0 and then decreased rapidly, while the OD_620_ of SPY1Δ*lytA* increased to about 1.0 and could maintain for 36 h (Figure 2A). The SPY1 bacterial suspension became clear at 12 h, while the SPY1Δ*lytA* bacterial suspension was still cloudy (Figure 2B).

Gram stains of SPY1 and SPY1Δ*lytA* cultured in C + Y liquid medium for 4 h indicated the morphology of these two bacteria was similar: Gram-positive, spearhead-like and in pairs or short chain-like arrangements. After 12 and up to 36 h, SPY1 was not visible in stained preparations of culture medium. However, SPY1Δ*lytA* cultures were similar to the 4 h samples at these later times. Cultures of SPY1Δ*lytA* for 24–36 h varied to round and abnormally form (Figure 2C). Together, these data indicated that we had successfully inactivated the autolysin function of SPY1.

### *In Situ* Mineralization of SPY1Δ*lytA*

**Figure 3:**
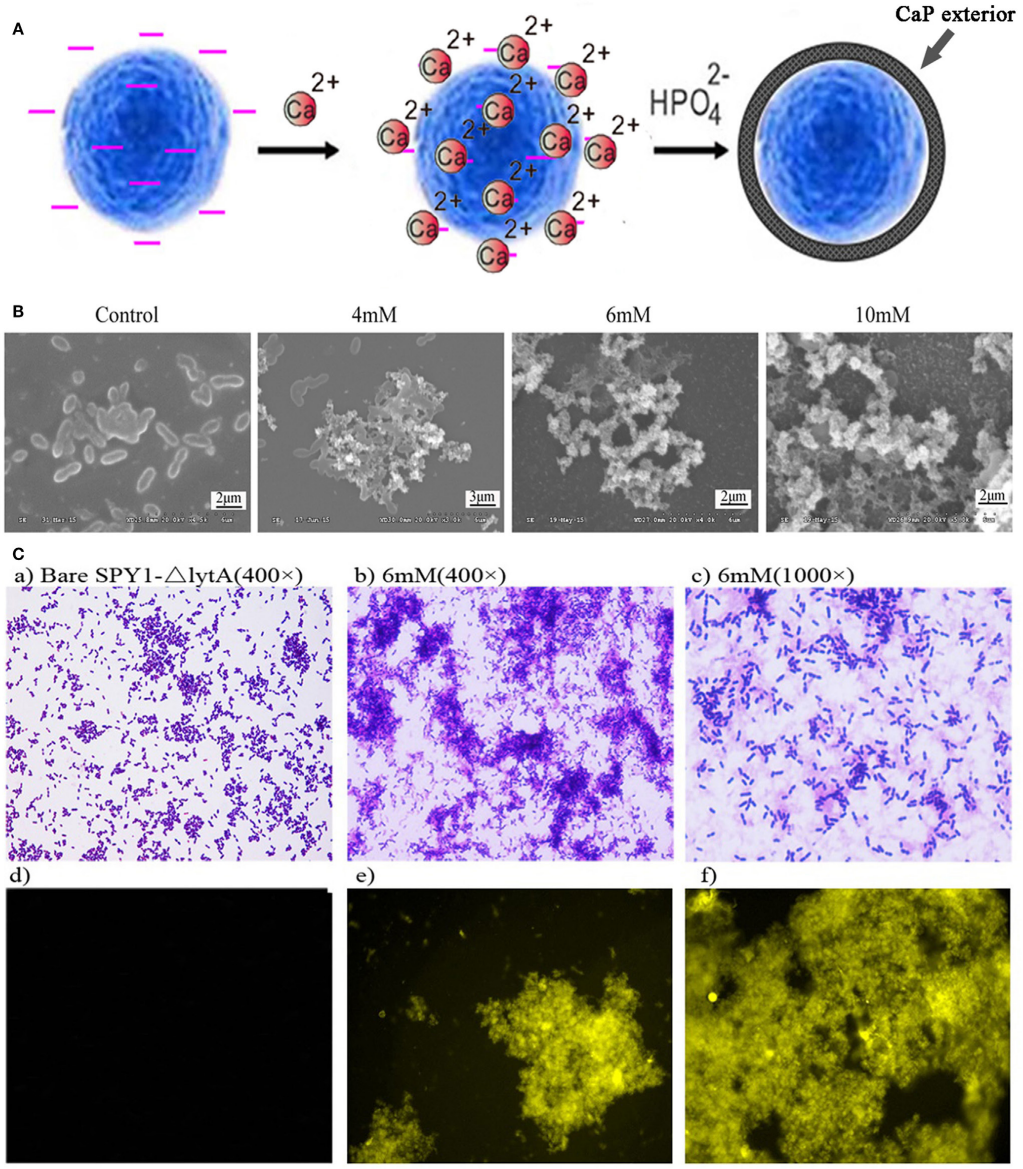
Construction of SPY1Δ*lytA*CaPi. **(A)** Schematic diagram of the *in situ* mineralization process of SPY1Δ*lytA*. **(B)** Scanning electron microscope images of CaP-mineralized SPY1Δ*lytA* after titration with phosphate solution in different concentrations. The native SPY1Δ*lytA* strain was used as control. **(C)** Mineral shells surrounding SPY1Δ*lytA*. (a–c) SPY1Δ*lytA* was stained with Gram staining. (d–f) the mineral shells was stained with tetracycline hydrochloride and observed under UV light (360–370 nm).

The bacterial strain SPY1 lacks capsule and therefore exposes other surface components such as teichoic acids. Zeta potential showed that SPY1 and SPY1Δ*lytA* present a net negative surface charge (−14.97 ± 0.75 and −15.2 ± 0.82, respectively), which enabled coating with calcium phosphate by *in situ* mineralization. The SPY1Δ*lytA* strain was treated with calcium-rich physiological saline to obtain a calcium-rich surface layer that acted as a nucleation site. Disodium hydrogen phosphate was then titrated into the calcium-rich SPY1Δ*lytA* suspension to obtain a CaPi mineralized surface layer (Figure 3A). SEM indicated that the optimum phosphate concentration for SPY1Δ*lytA*CaPi was 6 mM (Figure 3B). Tetracycline hydrochloride was used to characterize the calcium phosphate shell (Figure 3C), for it being able to bind with calcium ions to induce a golden-yellow fluorescence under UV light (360–370 nm). These results confirmed that SPY1Δ*lytA*CaPi was successfully constructed.

### The Mineralized Layer Improved the Thermal Stability of SPY1Δ*lytA* at 37°C

**Figure 4:**
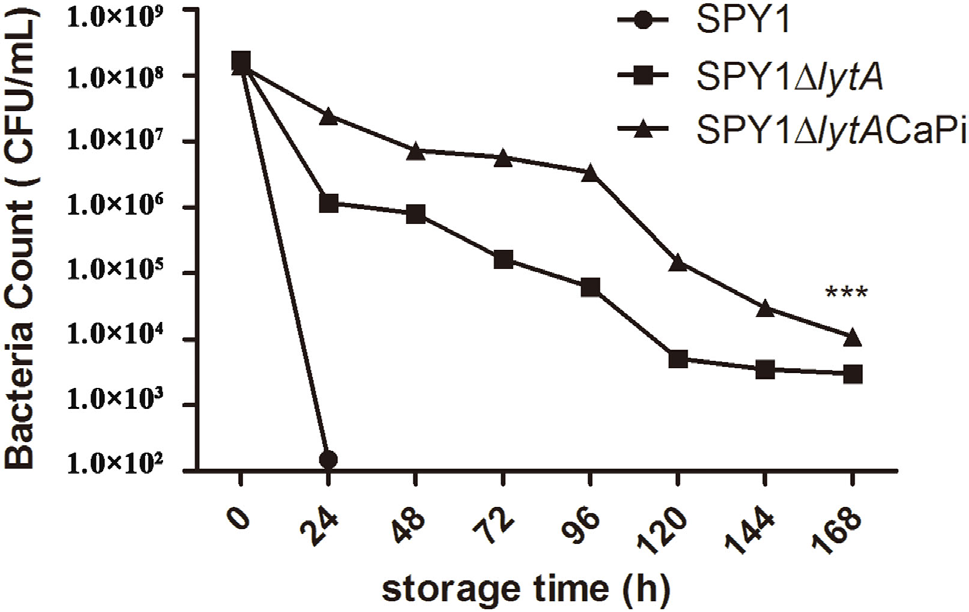
Thermal stability of SPY1Δ*lytA*CaPi. SPY1, SPY1Δ*lytA*, and SPY1Δ*lytA*CaPi were incubated at 37°C for 7 days, the concentration of bacteria was measured by CFU counting every 24 h. *P < 0.05, **P < 0.01, ***P < 0.001.

Thermal stability was determined by detecting bacterial viability at different time points after incubation at 37°C. The viability of SPY1Δ*lytA*CaPi was significantly greater than both SPY1 and SPY1Δ*lytA* (Figure 4).

### Vaccination with SPY1Δ*lytA*CaPi Induced Strong Immune Response in Mice

**Figure 5:**
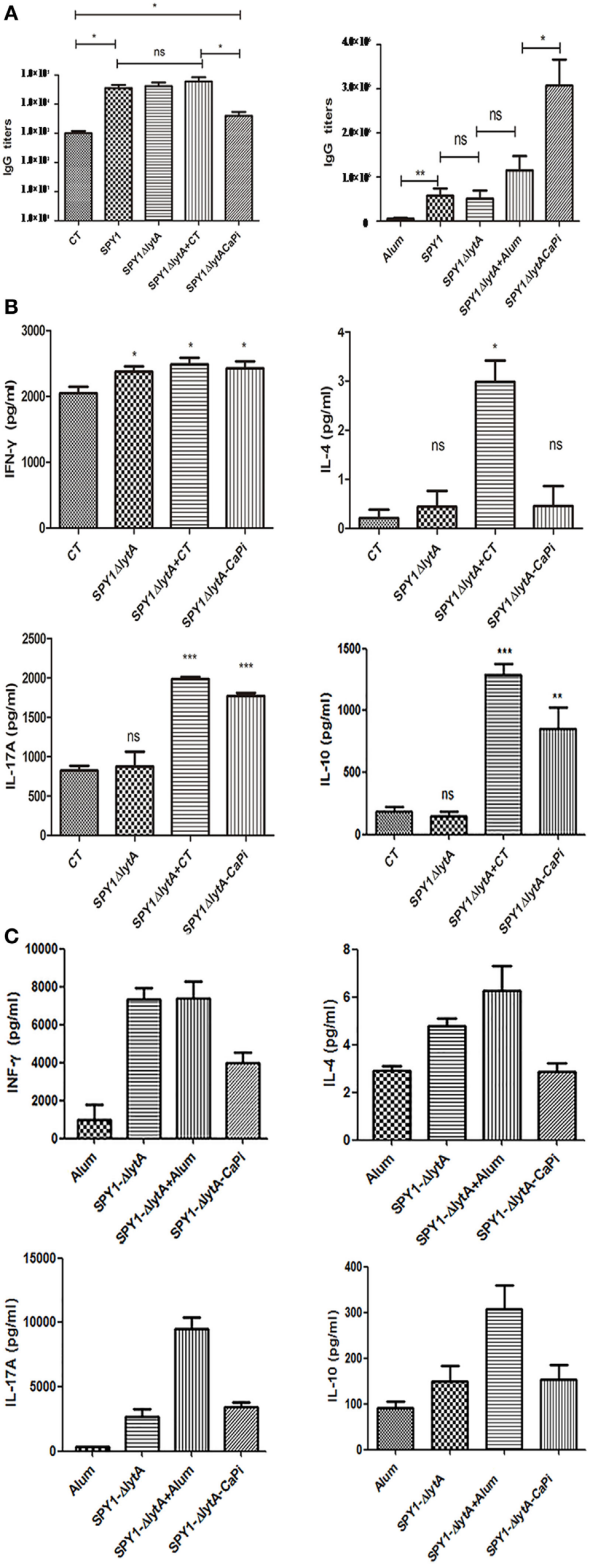
Humoral and cellular immune responses in mice induced by SPY1Δ*lytA*CaPi vaccine. For mucosal immunization, C57BL/6 mice were immunized with cholera toxin (CT), SPY1, SPY1Δ*lytA*, SPY1Δ*lytA* + CT, and SPY1Δ*lytA*CaPi, respectively. For subcutaneous immunization, C57BL/6 mice was immunized with alum adjuvant, SPY1, SPY1Δ*lytA*, SPY1Δ*lytA* + alum, and SPY1Δ*lytA*CaPi. **(A)** IgG titers in serum were measured 1 week after mucosal (left) or subcutaneous (right) final immunization. One week after the last immunization, suspensions of splenocytes (1 × 10^5^ cells/well) were cultured and exposed to 70% ethanol-killed SPY1 (equivalent to 10^7^ CFU/ml) for 72 h at 37°C. The levels of cytokine of splenocytes from **(B)** mucosal and **(C)** subcutaneous immunized mice were determined by ELISA kits. *P < 0.05, **P < 0.01, ***P < 0.001.

We examined the humoral and cellular immune responses of mice immunized mucosally and subcutaneously with SPY1Δ*lytA*CaPi. In subcutaneous-vaccinated mice, antigen-specific IgG levels of SPY1Δ*lytA*CaPi group were significantly higher than those of other groups. However, in mucosally vaccinated mice, IgG levels of SPY1Δ*lytA*CaPi group were similar to controls (Figure 5A).

In mucosally vaccinated mice, levels of IL-17A and IL-10 produced by splenocytes of mice immunized with SPY1Δ*lytA*CaPi were significantly higher than SPY1Δ*lytA* (Figure 5B). The elevation of cytokine in SPY1Δ*lytA*CaPi equals to which in SPY1Δ*lytA*CaPi + CT group, suggesting the mineralized vaccine strain had an adjuvant affect. However, the subcutaneous immunization of the mineralized vaccine strain SPY1Δ*lytA*CaPi did not result in elevated levels of IFN-γ, IL-4, IL-17A, and IL-10 (Figure 5C). These results suggested the immune responses induced by SPY1Δ*lytA*CaPi mucosal vaccination may be mediated by Th17 and Treg cells, while subcutaneous immunization stimulated humoral immune responses.

### Immunization with SPY1Δ*lytA*CaPi Reduced Pneumococcal Colonization

**Figure 6:**
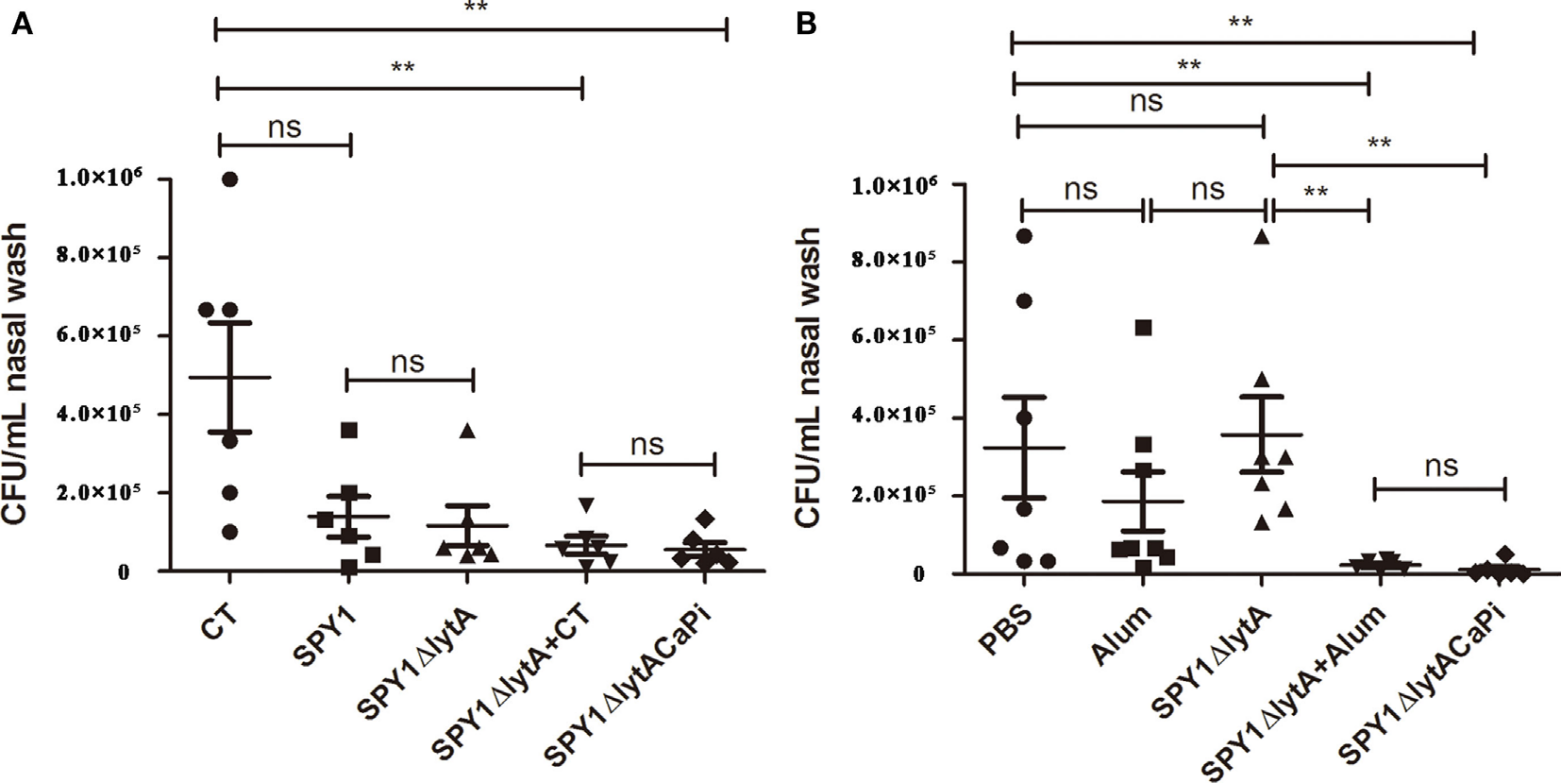
Immunization with SPY1Δ*lytA*CaPi reduced pneumococcal colonization. Mice mucosally **(A)** or subcutaneously **(B)** immunized with indicated antigens (mucosal with cholera toxin (CT), SPY1, SPY1Δ*lytA*, SPY1Δ*lytA* + CT, and SPY1Δ*lytA*CaPi, and subcutaneous with alum adjuvant, phosphate-buffered saline (PBS), SPY1Δ*lytA*, SPY1Δ*lytA* + alum, and SPY1Δ*lytA*CaPi) were intranasally challenged with *Streptococcus pneumoniae* serotype 19F (CMCC 31693, 1.5 × 10^7^). Bacterial loads in nasal washes were determined three days postinfection. *P < 0.05; **P < 0.01.

Nasopharyngeal colonization represents the initial step of invasive pneumococcal infection (26). The bacteria loads of mice mucosally or subcutaneously immunization challenged intranasally with *S. pneumoniae* strain 19F were measured by CFU counting. Nasal CFU counts in the SPY1 and SPY1Δ*lytA* vaccinated mice groups were not significantly different, which indicated that the deletion of the LytA protein did not affect the immunogenicity of SPY1. The bacterial loads for the mineralized vaccine SPY1Δ*lytA*CaPi group were significantly lower than the non-mineralized vaccine SPY1Δ*lytA* group, while it is similar to which of non-mineralized vaccine groups in the presence of adjuvants (SPY1Δ*lytA* + CT or SPY1Δ*lytA* + alum) (Figures 6A,B). These results suggested that the mineralized shells had adjuvant effects both in mucosal and subcutaneous vaccinations and that mineralized strain vaccination reduced pneumococcal colonization.

### Immunization with SPY1Δ*lytA*CaPi Provided Protection against Pneumococcal Lethal Infections

**Figure 7:**
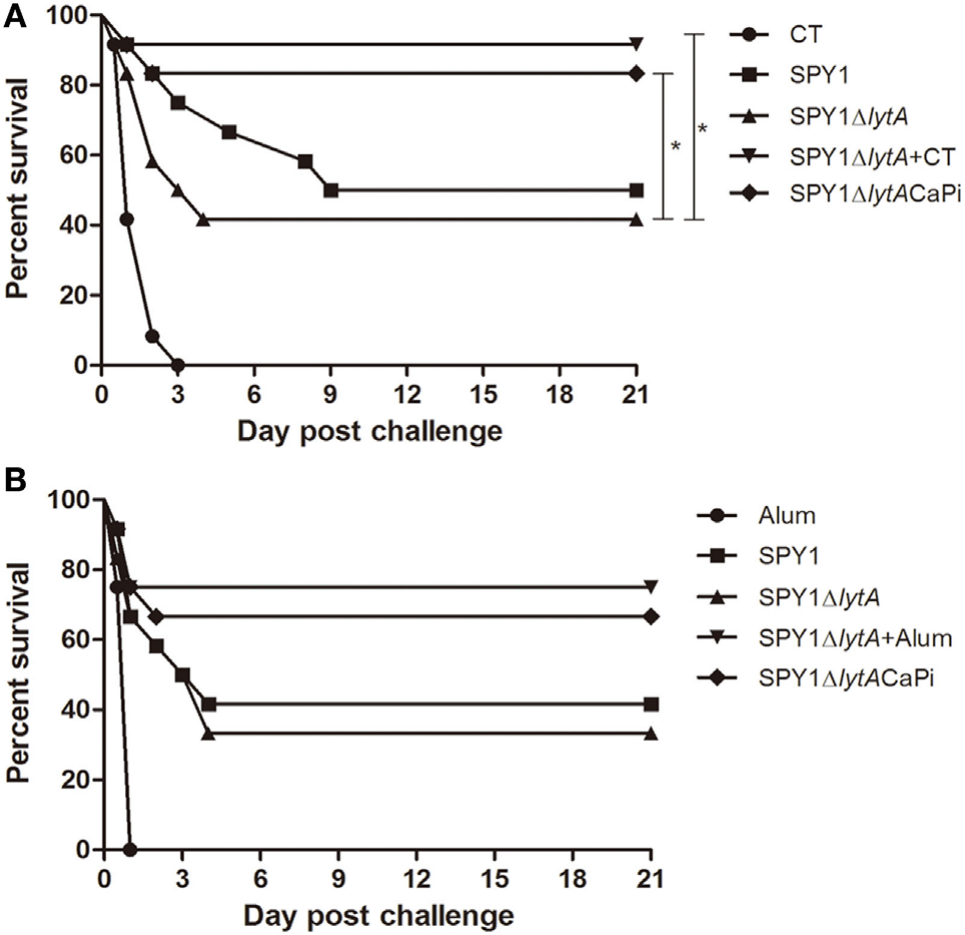
Protection against bacterial lethal infections by SPY1Δ*lytA*CaPi strain. C57BL/6 mice were mucosally **(A)** or subcutaneously **(B)** immunized with indicated antigens (mucosal with CT, SPY1, SPY1Δ*lytA*, SPY1Δ*lytA* + CT, and SPY1Δ*lytA*CaPi, and subcutaneous with alum adjuvant, SPY1, SPY1Δ*lytA*, SPY1Δ*lytA* + alum, and SPY1Δ*lytA*CaPi) and intranasally challenged with 1 × 10^8^ CFU of the D39 strain 2 weeks after last immunization. Survival was monitored for 21 consecutive days. The results were analyzed by log-rank test. *P < 0.05.

To evaluate the protective effects of SPY1Δ*lytA*CaPi vaccination against lethal infection of pneumococci, mice were challenged intranasally with *S. pneumoniae* strain D39 at 1 × 10^8^ CFU/mouse 2 weeks after last immunization. The mice survival rates for mucosal vaccination using SPY1Δ*lytA*CaPi were significantly higher than that of SPY1Δ*lytA* (Figure 7A). Similarly, the mice survival rates for subcutaneously vaccination using SPY1Δ*lytA*CaPi were higher than that of SPY1Δ*lytA*, but did not reach statistical significance (Figure 7B). In short, the mineralized strain vaccination could provide a better protective effect against pneumococcal infections than the non-mineralized strain.

## Discussion

SPY1 is a live attenuated *S. pneumoniae* strain and a vaccine candidate strain because it can elicit strong protective effects against *S. pneumoniae* challenges in mice (9, 10). However, the autolysin of the strain prevents its growth to the end of the logarithmic growth phase. This property limits the potential of SPY1 as a vaccine strain. We therefore inactivated *lytA* and the strain SPY1Δ*lytA* maintained unbroken cell shapes for an extended period indicating the autolysis property was eliminated. This made the strain more stable for storage and transport than the parent SPY1.

The instability of the vaccine products and inactivation of vaccines during storage and transportation have seriously hampered their effectiveness. The strain SPY1Δ*lytA* also needed to be cryopreserved. Mineralized vaccines can be stored at 37°C for more than 7 days and can stimulate higher antibody production after immunization (19). Therefore, we intended to improve the thermal stability and immunogenicity of SPY1Δ*lytA* by mineralization treatment.

The surface of SPY1 lacks a capsule and surface components such as teichoic acid are exposed. When SPY1Δ*lytA* is cultured in an alkaline environment, it has a net negative surface charge. We initially treated SPY1Δ*lytA* with a calcium-rich physiological saline to obtain a calcium-rich surface layer that could act as a nucleation site. We then determined the optimal phosphate to add to the suspension to obtain a CaPi mineralized surface layer. This resulted in a greater thermal stability for the mineralized vaccine than the non-mineralized strain. In addition, this procedure improved the immunogenicity of SPY1Δ*lytA* and mineralization induced high titers of IgG indicating the CaPi mineralized shell had also a good adjuvant effect. This means additional adjuvant such as CT is no longer needed. CT is toxic for humans and can not be used in human body, while CaPi is a component of human teeth and bones and had good biocompatibility and is easily absorbed (27, 28), and these are qualities for a good adjuvant.

We evaluated the immune protective mechanism of the SPY1Δ*lytA*CaPi mineralization vaccine in terms of humoral and cellular immunity. In mice vaccinated subcutaneously, IgG titers in the SPY1Δ*lytA*CaPi group were higher than those of the SPY1Δ*lytA* + alum group. This suggested that SPY1Δ*lytA*CaPi not only induces an effective humoral immune response in subcutaneous-vaccinated mice, but the mineralized components of the mineralized strain have an adjuvant effect. However, our experimental results in mucosal vaccinated mice showed that SPY1Δ*lytA*CaPi did not increase IgG level, this most likely due to the different modes of immunization.

In addition to humoral immunity, cellular immunity also plays an important role in antimicrobial infection. Proinflammatory cytokines such as IFN-γ, IL-4, IL-17A, and IL-10 play important roles in the immunization process against *S. pneumonia* (29–34). Our results indicated that SPY1Δ*lytA*CaPi significantly promoted the secretion of IL-17A and IL-10, compared with SPY1Δ*lytA* in mucosally vaccinated mice, suggesting that it could induce Th17 and Treg cell immune responses. However, IL-4 was not significantly induced in mice after immunization with mineralized strains, but IL-10 was elevated significantly. Because IL-10 is also a Th2-type cytokine as is IL-4, this indicated that the mineralized vaccine might also induce Th2 cell immune responses. However, there was no significant difference between SPY1Δ*lytA*CaPi and SPY1Δ*lytA* + CT group suggesting the mineralized layer creates an adjuvant effect and can activate Th17 and Treg cell immune responses.

We have also studied the protective effect of SPY1Δ*lytA*CaPi on nasal colonization of *S. pneumoniae*. Th17 cellular immune response plays an important role in this type of colonization by *S. pneumoniae* (34–36) and IL-4 is not involved (34). The results of our experiments showed that the bacterial loads in the nasal cavities of mice immunized with SPY1Δ*lytA*CaPi were significantly lower than that of the non-mineralized vaccine group (*P* < 0.01). Mechanistically this may be due to a greater Th17 cell immune response after mucosal immunization. Secretion of IL-17A into the nasal cavity recruited neutrophils that effectively removed the bacterial infection (37).

In our study, we found that the colonization effect of SPY1Δ*lytA*CaPi group bears comparison with non-mineralized vaccine groups in the presence of adjuvants (SPY1Δ*lytA* + CT or SPY1Δ*lytA* + alum). Familiar phenomenon was also observed in survival rates evaluation. These results suggested that the mineralized shell had an adjuvant effect. In addition, in mice vaccinated mucosally, the survival rates of the SPY1Δ*lytA*CaPi group were significantly higher than those of SPY1Δ*lytA* group, which were consistent with the results of cytokines. This indicated that mucosally vaccinated the mineralized strain could provide better protection against pneumococcal infections than the non-mineralized strain in mice.

In summary, we constructed a mineralized bacterial strain SPY1Δ*lytA*CaPi and evaluated its thermal stability, immunoprotective effects, and immune protection mechanism. The mineralized shell increased bacterial stability. The protect effects of mineralized vaccine were superior to non-mineralized vaccine both in mucosal and subcutaneous vaccinations. The former induced mainly Th17 response while the later increased IgG levels. The results of this study lay the foundation for further research and development of *S. pneumoniae* vaccines.

## Ethics Statement

All the animal experiments were done in accordance with the guidelines of the Institutional Animal Care and Use Committee of Chongqing Medical University.

## Author Contributions

WX and XZ designed the studies and wrote the paper. XZ and JC performed experiments about SPY1Δ*lytA* mutant strain construction and mineralization. YW, HW, and YQ performed animal experiments. JW and YM analyzed data. YH, XZ, and YY provided advice in experiments.

## Conflict of Interest Statement

The authors declare that the research was conducted in the absence of any commercial or financial relationships that could be construed as a potential conflict of interest.
